# Benefits and risks of health data reuse for healthcare providers: stakeholder perspectives from a qualitative interview study

**DOI:** 10.1186/s12913-025-12500-7

**Published:** 2025-03-18

**Authors:** Susanne Stark, Susanne Gabriele Schorr, Merle-Marie Pittelkow, Daniel Strech

**Affiliations:** Berlin Institute of Health at Charité - Universitätsmedizin Berlin, QUEST Center for Responsible Research, Berlin, Germany

**Keywords:** Health data, Data sharing, Secondary use, Healthcare provider, Stakeholder perspective, Risk, Risk mitigation, Reasonable decision-making

## Abstract

**Background:**

Reusing health data, for example for research into the quality of care or healthcare planning, has far-reaching potential. Current ethical discussions on developing health data platforms (e.g. the German Medical Informatics Initiative, MII) have primarily addressed patient-related benefits and risks of reusing this data. Less is known about the perspectives of healthcare providers, such as hospitals, that make health data available through these platforms. However, the risks they perceive and a resulting reluctance to share data, along with the lack of strategies for managing these risks, could significantly impede research with health data. In this exploratory qualitative study, we investigated the interests and risks relevant to healthcare providers in the secondary use of health data, and strategies to address these risks (pre-registration: https://osf.io/uxds).

**Methods:**

We conducted online expert interviews (*N* = 21) between May and August 2023 with German representatives of different stakeholder groups (e.g. healthcare providers, researchers, patient representatives, data protection officers) with expertise in the secondary use of health data and the associated interests and risks for providers. We analysed the data using the deductive-inductive approach to qualitative content analysis.

**Results:**

Interviewees attributed several potentials reusing health data, such as improving healthcare quality and transparency. They also pointed to risks, including their causes and consequences for providers’ reputation, economic and existential situation. Risks included a) biased results and interpretation of data analyses due to inadequate data validity and inappropriate analytical approaches, b) questionable reuse purposes**,** c) liability risks due to a lack of control over shared data and unresolved legal questions, and d) improved transparency that may reveal challenges and misconduct in healthcare. Suggested strategies for risk mitigation related to use and access decisions on secondary use requests. These include a) developing risk-reducing policies, b) ensuring transparent decision-making processes by involving all relevant stakeholders and applying structured risk–benefit assessments, and c) measures to improve the validity of secondary analyses. In addition, the interviewees identified further need for action to be addressed by providers, researchers and policymakers.

**Conclusion:**

These findings point to needs and opportunities for action to manage risks that providers associate with health data reuse. Decision-making processes on secondary use requests should be based on normative principles, and quality, safety and trust in health data reuse should be enhanced. These recommendations should be taken up by responsible stakeholders in initiatives such as the MII, among researchers and policymakers to reduce reluctance and promote research with health data.

**Supplementary Information:**

The online version contains supplementary material available at 10.1186/s12913-025-12500-7.

## Background

The secondary use of health data has far-reaching potential for improving healthcare [1–3]. Initiatives such as the European Health Data Space (EHDS) [4] or the German Medical Informatics Initiative (MII) [5] aim to improve access to and the reuse of data from (electronic) health records, health insurances, or registries for research and development, referred to as secondary use. To achieve this, the main objectives of these initiatives include developing secure and interoperable infrastructure and standards for efficient data management, as well as ensuring data quality, validity and security [6, 7]. Another important task is to ensure compliance with legal and ethical principles for data sharing and reuse, e.g. obtaining informed consent and protecting data donated by patients [6–8].

At present, the scientific discourse around the secondary use of health data has focused primarily on patients’ willingness to share health data, their interests, perceived potentials and risks associated with secondary use [9–12]. Less is known about the interests of healthcare providers (e.g., hospitals, nursing homes, medical practices) – especially in Germany. However, in initiatives like the MII, healthcare providers play an important role in enabling health data reuse. They are core members of Use and Access Committees (UACs, also known as Data Access Committees), which are responsible for reviewing and deciding on secondary use requests and granting access to institutional health data. In the MII, UACs belong to Data Integration Centres (DIC) as institutional parts of University Medical Centres, in which health data are made accessible for reuse. UACs consist of members representing at least the institutional management of the certain healthcare provider, the data management (DIC), legal (data protection officer) and methodological (epidemiology/biometrics) expertise. Other experts (e.g. ethicists, medical specialists) may be involved [13]. However, there are various ways in which Data Access Committees are established, constituted and operated [14].

There is evidence indicating that decisions on secondary use requests can depend on the willingness of individual providers to share data. This willingness can in turn be influenced by perceived potentials and risks associated with a reuse request. For example, studies show that openness to data sharing may be driven by perceived benefits to individual patient care, decision-making, or healthcare quality [15–17]. In contrast, concerns may lead to reluctance to share data. Such concerns may arise from perceived risks associated with unauthorized access to and misuse of shared data [16–20], poor quality, analyses or interpretation of secondary data [18], or competitive and reputational disadvantages caused, for example, by disclosure of sensitive business information or poor quality of care [15, 18–21].

Strategies to address such concerns and protect the legitimate interests of healthcare providers, without compromising the potential of research with health data, are rarely addressed in the scientific discourse. Measures that are occasionally proposed include considering and assessing risks in use and access decisions [17, 19, 22], restricting the reuse purposes [18, 23], or ensuring privacy protection and security [16, 17, 23]. Risks and their management in decision-making processes are also rarely addressed in publicly available use and access policies [24]. For example, the national MII policy defines objective decision criteria like the scientific quality of secondary use requests or the scientific expertise of applicants [13]. However, these criteria do not include risks that healthcare providers may face when sharing data for research purposes, nor measures to mitigate these risks. Consequently, it remains unclear, whether and how risks are considered in UACs and what impact they might have on decision-making processes.

Considering the interests of healthcare providers is of great importance. Without a differentiated assessment of their interests, they may be under- or overestimated, ultimately limiting the benefits of research with health data. Conversely, targeted risk mitigation strategies can help to reduce healthcare providers’ reluctance to share data, and to support research.

Therefore, this study had two objectives. First, we aimed to investigate the interests that healthcare providers in Germany may have when sharing health data for secondary use, focussing on the perceived risks, their characteristics and relevance. Second, we aimed to identify approaches to risk mitigation that could simultaneously promote the reuse of health data.

## Methods

### Ethics approval and preregistration

The study received approval from the Ethics Committee of Charité – Universitätsmedizin Berlin, Germany (No.: EA4/067/22). Study documentation and reporting were based on the COREQ checklist [25] (Suppl. 2). The study was preregistered on the Open Science Framework (10.17605/OSF.IO/UXDSA). Deviations from the original study design, a reflection and positioning of the study team are available in Supplements 1 and 3.

### Study design

An exploratory qualitative design was adopted. We conducted semi-structured expert interviews to investigate stakeholder perspectives on our research topic. This approach is suitable for in-depth exploration of perspectives of participants with exclusive knowledge of a topic or problem and its relevance, including specific approaches to decision-making and problem management [26]. We analysed the data material using qualitative content analysis, which involved deductive and inductive coding. This approach is suitable for systematically gathering and comprehensively describing interviewees’ perspectives and understanding of the research topic [27].

### Sampling

We defined experts as individuals with exclusive knowledge and access to information [26] on the interests and risks for healthcare providers associated with the secondary use of health data. We applied a purposive sampling strategy to identify eligible individuals across Germany. We predefined sampling criteria (stakeholder groups and their expertise) to include persons who could contribute to exploring the topic from different perspectives. We sought to identify experts with knowledge and experience of the roles and interests of healthcare providers in the context of health data sharing and secondary use for research purposes, the research with health data that may affect specific provider interests (e.g. aims, requirements, practices), and the political, legal, technical, and ethical requirements and issues involved. We expected representatives of the following stakeholder groups to have expertise in one or more of these aspects: 1) healthcare providers with experience in data sharing for research purposes, 2) scientists requesting and using health data for research purposes, 3) patient representatives as advocates of data owners (patients), 4) data protection officers, 5) persons responsible for regulating, implementing, and managing the reuse of health data, 6) self-governance bodies in the German health system. Based on these criteria and drawing on the study team’s knowledge of the field, one team member (ST) first identified and mapped eligible individuals through an online search of relevant German institutions and organisations. We discussed and prioritised potential interviewees in the study team.

### Recruitment

Based on our sampling criteria, we recruited participants between March and May 2023. We recruited additional participants between June and July 2023, which we included based on recommendations from interviewees (snowball sampling) provided they met our sampling criteria. We invited Individuals via email, which included a link to the study protocol, the study information, and the consent form (see Suppl. 4). We offered participants a compensation of €150. We followed up by email after one week and by phone after two weeks if we did not receive a response to the initial invitation. After participants agreed to be interviewed, we scheduled an online interview via Microsoft Teams. We stopped recruiting when empirical saturation [28] occurred, defined as the point at which collecting and analysing new data did not yield new insights. We made this decision based on reflection of the interviews, their documentation, and the iterative analysis process.

### Data collection and processing

We conducted semi-structured expert interviews [26] based on an iteratively developed interview guide. First, we drafted a preliminary guide based on a literature search and background interviews that were conducted prior to the study (see https://osf.io/uxdsa), which we then piloted and revised in team discussions. The resulting interview guide, which can be found in Supplement 5, covered the three thematic dimensions of our research questions (Table 1) and was used flexibly depending on the individual interview situation. All interviews were conducted and recorded via Microsoft Teams. Apart from the researchers and participants no other persons attended the interview. We did not conduct repeat interviews. One team member (ST) led the interviews and another team member (MMP, SGS, or DS) was responsible for organisational aspects and postscript writing. We narratively checked the accuracy of the information obtained and the adequacy of our understanding during the interviews by summarising, paraphrasing and discussing the interviewees’ statements. Each interview was followed by a peer-debriefing on the atmosphere and dynamics of the conversation, the thematic focus and methodological peculiarities. We documented our impressions in structured postscripts (Suppl. 6). The recordings were fully transcribed by an external company using a basic transcription system [29]. All transcripts were subsequently pseudonymized prior to data analysis.

**Table 1:** Thematic dimensions of the interview guide

Core dimension	Subthemes
1. Interests of healthcare providers associated with the secondary use of health data	
2. Risks for healthcare providers associated with the secondary use of health data	• Characteristics and consequences of perceived risks
	• Risks in the context of research questions and studies
	• Risk–Benefit-Assessment
3. Context/strategies to minimize risks and facilitate research	• Status quo assessment
	• Future needs for further development

### Data analysis

We used a deductive-inductive approach to qualitative data analysis [27]. The main categories of the coding tree were defined from the core themes of the interview guide (Table 1) and subcategories were developed inductively from the data. Using MAXQDA 2022 [30], one team member (ST) developed a preliminary coding tree based on one interview transcript from each stakeholder group (*n* = 6). Two other members of the study team (SGS, MMP) independently piloted this coding tree using the same material. Coding and interpretation discrepancies were resolved through group discussions, and the coding tree was revised and refined based on consensus. ST then coded the entire material based on the revised coding tree. Throughout this process, minor adjustments (differentiation or titles of categories) were made without developing new (sub-)categories. We discussed and agreed on these changes within the research team. We then summarised the coded material across cases and stakeholder groups, taking into account similarities and differences in perspectives. For the final coding tree please see Supplement 7.

## Results

###  Recruitment and sample

We invited a total of 29 individuals; 21 agreed to participate in an interview. Nineteen of the interviewees were recruited through purposive sampling and two through snowball sampling. We conducted interviews between May and August 2023. The interviewee and interview characteristics are presented in Table 2.

**Table 2:** Interviewee and interview characteristics

Attribute		*n*
Interviewees (*N* = 21)		
Sex	Female	8
	Male	13
Stakeholder group	Healthcare providers (L)^a^	6
	Self-governance bodies (IV)	3
	Scientists (F)	6
	Data security officers (D)	3
	Patient representatives (P)	3
Responsible for regulation, control or implementation of secondary use	Yes	9
	No	11
	Unknown	1
Prior relationships between researchers and interviewees	Yes	6
	No	15
Interviews (*N* = 21)		
Setting	Web meeting	21
Duration, min.; Ø (min, max)	Total	57:49 (43:00, 75:00)
	recording	48:44 (34:42; 58:55)

^a^Letters in brackets: Stakeholder group identifier used for quote citation

**Fig. 1:**
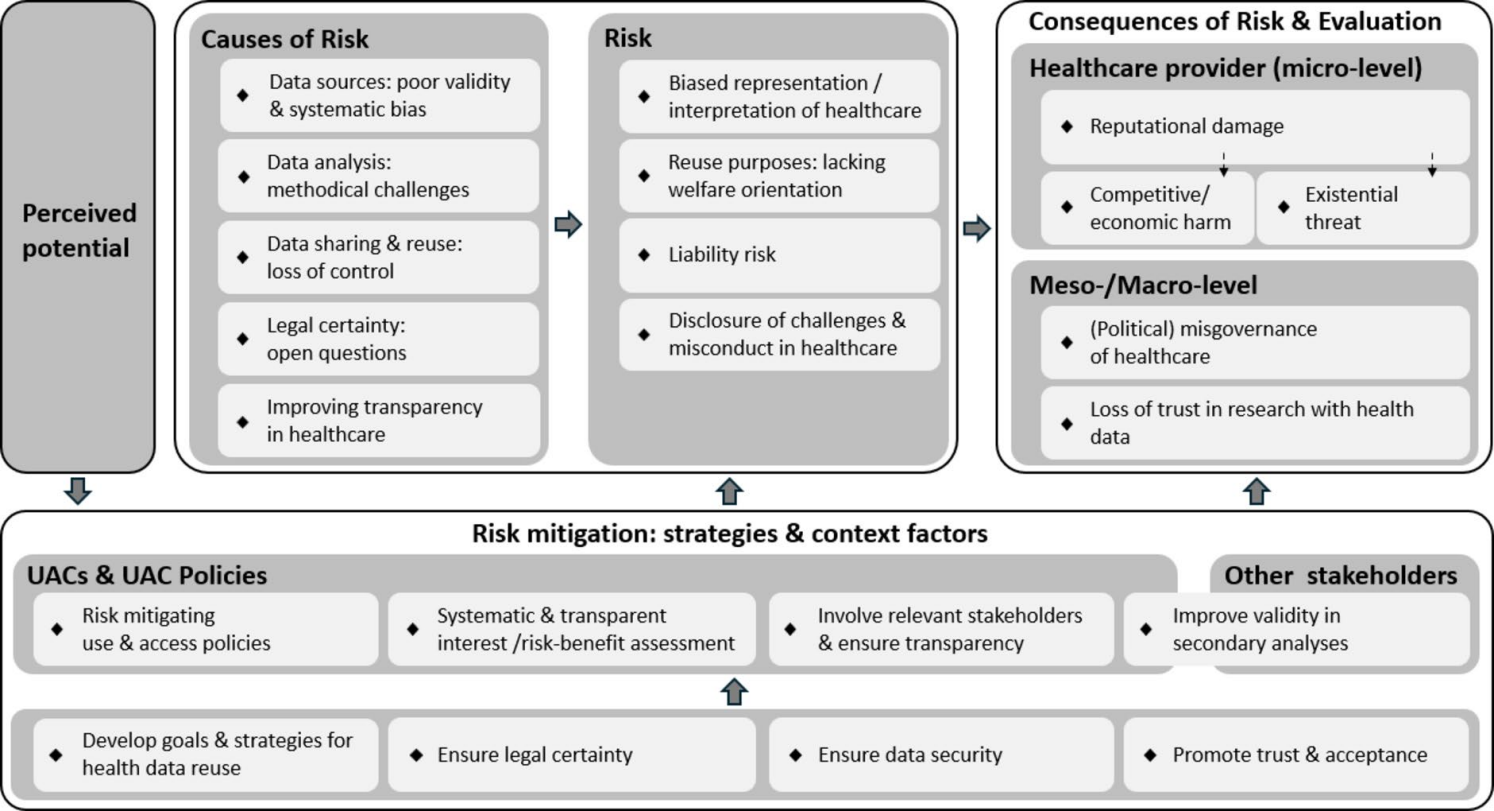
Main findings: Overview of main coding categories, subcategories and interrelations UAC: Use and Access Committee

Figure 1 provides an overview of the main findings based on the final coding tree we developed. It shows the main thematic categories (i.e. perceived potentials, risks and their characteristics [causes, consequences, evaluation], and risk mitigation strategies in the secondary use of health data), the corresponding subcategories and their interrelations. We summarise and specify these findings below.

### Perceived potentials

Overall, interviewees from all stakeholder groups saw a variety of potentials for healthcare providers associated with the secondary use of health data. These ranged from improving the performance and quality of healthcare, advancing needs-based approaches to healthcare, improving transparency and reducing deficiencies in healthcare, to closing knowledge gaps. However, as the following quote illustrates, some participants pointed out that these potentials are primarily recognised by providers who are (in-)directly involved and experienced in health data reuse, i.e. mainly the medical profession involved in existing initiatives such as the MII. Other providers (e.g. allied health professionals, outpatient providers) may lack experience and therefore awareness of potential benefits.

*"[…] they [university medical centres] always have a dual role. On the one hand, they hold the provider role, but of course also the role of researcher. So, one must be a little bit careful not to take them [providers] pars pro toto. The ordinary provider, I would say, is my general practitioner, and the situation there is, of course, completely different." (I-09D, pos. 10)*

### Perceived risks

The interviewees emphasised that the secondary use of health data can be associated with risks and consequences for healthcare providers that can lead to reluctance to share data for research purposes.

- a) Causes of risks

We identified five main causes of perceived risks (data sources, data analysis, data sharing and reuse, legal questions, improved transparency, see Fig. 1) from the participants’ reports.

The causes related to limitations inherent to the *data sources* (e.g. lacking granularity, documentation quality, and validity, systematic bias) and *data analyses* (e.g. inadequate data processing and analytical approaches) are evaluated as substantial threats.

*“Secondary data in the hand of the ignorant (...) is decidedly dangerous.” (I-01F, pos. 11)*

According to the participants, these threats primarily arise from a lack of knowledge about the specific characteristics of the data sources, insufficient skills in the selection and evaluation of variables, indices and analytical procedures, and inappropriate interpretation of results from secondary data analysis. In some instances, interviewees suggested that data users might lack a sense of responsibility resulting in the use of questionable research practices (e.g. p-hacking).

Another concern of providers reported by the interviewees is the potential *loss of control over shared data and reuse* purposes. It is closely related to *unresolved legal questions* as another risk-causing factor:

*“(…) what certainly discourages [data sharing] is the fear of what will happen to this data. (...) that the data will disappear into whatever channels and that sinister things will be done with it (…).” (I-05F, pos. 14)*

Although this fear was rejected by some of the participants as a common narrative of data sharing opponents, others considered the underlying concerns to be legitimate due to legal uncertainties. For example, some participants considered the effectiveness of limiting reuse purposes and monitoring rules for data users (e.g. researchers, health insurers, pharmaceutical industry) in existing data use agreements to be insufficient. In their view, this could lead to data sharing or reuse for unauthorised purposes.

In addition, improved *transparency in healthcare* was perceived as a potential source of risk, although it was also seen as one of the benefits of health data reuse. Interviewees argued that while research can help improve the evidence base on the processes and quality of healthcare, it could also have undesirable effects on providers, e.g. by increasing their accountability to the public or competitive dynamics.

- b) Risks

Four main types of risks were discussed in the interviews: a) biased representation and interpretation of healthcare, b) questionable reuse purposes, c) liability risks and d) disclosure of challenges and misconduct in healthcare (see Fig. 1).

When discussing *biased representation and interpretation* of healthcare, the interviewees referred to inaccurate analyses and reporting in the reuse of health data. As illustrated by the following quote, these biases are primarily considered unintentional and attributed to the limitations of data sources and analytical approaches:

*“If there is risk selection in hospitals at the expense of tertiary care providers, yet the corresponding measuring tools fail to adequately reflect this risk situation, certain [hospital] structures will always look better than others.” (I-21L, pos. 29)*

However, some participants also pointed to the risk of deliberate misinterpretation by stakeholders (e.g. providers, health insurers, political representatives) to exert (political) influence.

Furthermore, participants emphasised the risk of *questionable reuse purposes* resulting from insufficient control over shared data and legal uncertainty. Questionable in this context meant:

*“(…) that data are used, for example, to serve or satisfy business models rather than patient oriented.” (I-14IV, pos. 9)*

Such purposes included, for example, purely commercial interests (e.g. customer acquisition, product marketing), which interviewees argued are not oriented towards the public good or patients. Scepticism was also expressed towards reuse purposes that may appear patient-oriented but could be influenced by questionable individual interests, such as data-driven patient navigation by health insurers with the primary aim of cost saving.

Interviewees emphasised that unauthorised sharing and reuse by data users may involve *liability risks* for healthcare providers who have granted access to data. Considering the grey market for health data, some participants judged these risks to be highly relevant in the context of inadequate control over shared data and legal uncertainty.

Risks described with regard to increased transparency through health data reuse comprise the undesired *disclosure of challenges* (e.g. treatment errors, over- or undertreatment, lacking guideline adherence) *or misconduct* (i.e. fraud, corruption) in healthcare.

*“(…) then, of course, many physicians are also afraid that now things will be uncovered that they aren’t doing well.” (I-06L, pos. 16)*

These may also be associated with liability risks, for example, if criminal offenses are uncovered or wrongly raised (e.g. due to inadequate data analysis). Although the interviewees emphasised that the latter cases are to be avoided, they generally assessed the benefits of improved transparency to outweigh providers’ risks of uncovering and addressing actual issues in healthcare, as the following quote referring to patient safety illustrates:

*“What we are currently experiencing out there every day is a considerable risk for patients in particular (...). That is to say, a fundamental increase in transparency is, in my view, rather risk-reducing.” (I-01F, pos. 27)*

- c) Consequences

The interviewees considered the consequences resulting from the reported risks from different perspectives. They refer to consequences for a) individual providers at the micro level, and b) provider groups, healthcare regions or the health system at the meso/macro level (see Fig. 1).

The main consequences discussed at the micro level were loss of reputation, competitive and economic disadvantages, and existential threat. *Reputational damage* was primarily discussed in the context of disclosure of actual or supposed challenges and misconduct in healthcare, which could subsequently have *competitive, economic and existential consequences*. For example, participants explained that the disclosure of quality deficiencies by a particular provider can lead to a loss of patients and ultimately to economic harm. The interviewees also stressed that competitive, economic and existential harm could result from the disclosure of sensitive business information (e.g. on patents, turnover and revenue), data-driven healthcare planning and management (e.g. restructuring of cost-relevant service areas), and quality assurance (e.g. minimum quantity regulations. The relevance and severity of individual consequences are subject to the interviewees’ risk–benefit assessment. One of the key criteria applied for this is the benefit of research with health data for society and the common good. Interviewees emphasised that potential consequences must be accepted as long as the benefits (e.g. revealing and addressing healthcare quality/patient safety issues) outweigh the harms to individual providers.

*“As far as it concerns the fear of healthcare providers that [research] will reveal something of poor quality or so, this is definitely secondary to me. We have to tolerate that (…). Well, these are things that are absolutely important for the patients. But of course, it's a shock for the hospitals when it says: "Well, a hospital with less than fifty something procedures will no longer be allowed to do that in future. But it is definitely, it is absolutely ethically justifiable and, in my view, this has to be done and if you recognise these associations, they of course have to be implemented.” (I-06L, pos. 18)*

In contrast, the participants unanimously argued that legitimate protection interests of providers must be assured, and consequences avoided, if secondary analyses are misleading, if allegations are wrongly raised, or if they have no societal benefit.

For some of the participants, an additional criterion in weighing potential harms was the nature of (public) reporting of secondary analyses. They argued that publications that adhere to scientific standards or are set within a professional context (e.g. quality assurance) are associated with a lower potential for harm than, for example, media releases or information on patient portals that lack (adequate) quality standards.

At the meso- and macro level, interviewees pointed to consequences for data-driven healthcare governance and regulation, and the trust in research with health data. Both issues were seen as serious and should be avoided. First, they argued that although health data reuse holds vast potential for the further development of healthcare, it also carries the risk of *(political) misgovernance* (e.g. misleading resource planning) if the limitations of data sources and methodological issues are not adequately considered, and results are not trustworthy.

*“(…) that simply wrong conclusions are drawn (...), which then lead to interventions at the political level or to incentive structures that trigger misgovernance.” (I-21L, pos. 29)*

Secondly, these risks and questionable reuse purposes could result in a *loss of societal trust in research with health data*. This consequence was particularly emphasised by participants from research, healthcare providers, and self-governance bodies.

*“THAT, in turn, I think is the biggest risk, also for non-commercial research, that the good purposes and intentions are damaged." (I-18IV, pos. 16)*

The interviewees attributed the severity of this consequence to the increasing scepticism of patients and society towards the establishment and expansion of data-sharing infrastructures and reuse.

### Risk mitigation strategies

The risk mitigation strategies discussed related to a) the decision-making processes on access to and secondary use of health data in bodies such as UACs, and b) improving the validity of and contextual factors for secondary analyses of health data.

- a) Improving use and access decisions

According to the participants, existing *use and access policies* should be complemented by provisions that they believe are often missing.

*“There should be general guiding principles that any reasonable use and access procedure should adhere to in order to be considered a reasonable use and access procedure.” (I-21L, pos. 83)*

Such additional provisions should include, for example, exclusion criteria for reuse based on public interest principles, and measures to prevent questionable reuse or misuse (e.g. by excluding certain stakeholders, introducing effective control mechanisms and sanctions).

A key risk mitigation strategy across interviews was the *systematic and transparent interests- and risk–benefit assessment* of secondary use requests. From the respondents’ perspective, this requires binding principles that allow for the assessment of a) the legitimate protection interests of all affected stakeholders, as well as the associated risks and potential harms, and b) the benefits associated with individual projects. These principles should include a focus on the public good, access to health data that is as independent as possible from particular interests, and a critical case-by-case assessment. According to the participants, the assessment principles and procedures need to be standardised and transparent to ensure neutrality and trustworthiness in the decision-making process.

*“(...) we must succeed in removing particular interests from the game as far as possible.” (I-21L, pos. 79)*

To achieve this, several key information should be publicly available. This includes information on the tasks and procedures of decision-making bodies, on secondary use requests, on the reviewers (including conflicts of interest) and on review results (approval, rejection, reasons). For this purpose, some interviewees suggested establishing a mandatory public registry, e.g. based on the MII's Medical Research Data Portal (Forschungsdatenportal Gesundheit, https://forschen-fuer-gesundheit.de/).

Another key issue discussed in the interviews was the *composition and independence of decision-making bodies*. With regard to the composition of decision-making bodies, interviewees emphasised that all stakeholder groups affected by secondary use projects should be represented and equipped with voting entitlement to ensure that their different interests and risks are equally taken into account. The stakeholder groups that, according to the interviewees, should be mandatory members of decision-making bodies, are listed in Table 3.

**Table 3:** Stakeholders who should be mandatory members of decision-making bodies and their roles

Stakeholder	Roles
Healthcare providers (different disciplines and professions) or their representatives	• Data provider
	• Involvement depending on individual secondary use requests
	• Technical, content-related and methodological review of requests
Representatives of patients / society	• Data donor
	• Assessment of public interest of secondary use requests
	• Corrective to provider- and other interests in the risk–benefit assessment
Scientists (different disciplines) or scientific organizations	• Scientific and methodological assessment of secondary use requests and risks
Data protection officer, ethicists	• Legal & ethical assessment of secondary use requests and data sources

Participants expressed diverse views on whether and how to assure neutrality and independence in decision-making processes. On the one hand, interviewees stressed that these are basic requirements for the trustworthiness of decision-making processes. On the other hand, representatives of healthcare providers in particular argued for balancing these principles with the protection of stakeholders' legitimate interests (e.g., safeguarding sensitive business information), although it remained vague how legitimacy should be defined. Similarly, different positions on where to anchor decision-making bodies became apparent. Several interviewees favoured a legally mandated statutory institution similar to a ‘permit authority’ (cf. e.g. Findata) to minimise the influence of particular interests and arbitrary decision-making. Other participants preferred to retain the existing decentralised structure (i.e. local UACs, e.g. at university medical centres), although acknowledging that this would be unlikely to reduce the influence of particular interests.

Finally, *improving the validity of secondary analyses of health data* was identified as an important aspect of risk mitigation. Interviewees stated that minimising the risks arising from the limitations of data sources and analytical methods would simultaneously enhance the potential of research with health data.

*“(…) we are not operating in the most highly standardised area of clinical research, (…) but rather we are seeing real life here, what then also has the chance to be perhaps somewhat more broadly generalised, if we actually succeed in overcoming these methodological pitfalls.” (I-10F, pos. 54)*

Interviewees assigned responsibility for implementing validity improvement strategies to different stakeholders. Table 4 provides an overview of the responsibilities of decision-making bodies, data providers (i.e. healthcare providers), policymakers and data users discussed.

**Table 4:** Interviewees’ perspectives on stakeholders and responsibilities for improving validity of secondary analyses of health data

**Decision-making bodies**	
	• Ensure methodological expertise of applicants
	• Ensure technical and scientific quality of reuse projects
	• Ensure consistent and transparent application of good scientific practices
**Data holders**	
** Improve data quality**	• Appropriate preparation and curation of data sources
	• Provide and harmonise metadata
	• Mandatory assessment of data quality
** Improve interoperability & linkage of data sources**	• Ensure high-quality data structure
	• Ensure interoperability of data sources
	• Harmonise different data inventories
**Data user**	
** Ensure research quality**	Promote expertise and quality in the secondary use of health data • Internal/external validation of data sources
	• Continuous evaluation and improvement of analytical methods
	• Promote research quality meta-analyses
	• Establish and expand systematic qualification and networking programmes
** Avoid misinterpretation**	• Ensure results reporting tailored to target groups
	• Promote responsible science communication
**Overarching responsibilities**	
** Improve data sources**	• Unlock additional data sources
	• Improve opportunities for data validation and linkage
	• Establish and expand infrastructure and methods
	• Facilitate data reuse across sectors and different legal bases
	• Obligate healthcare providers to share data
** Improve interoperability & linkage of data sources**	• Improve findability and usability
	• (further) develop secure legal opportunities for data linkage

- b) Improving context factors for the secondary use of health data

Participating researchers, healthcare providers, patient representatives and data protection officers highlighted the need for improving the context of health data reuse for risks mitigate. They called for the development of overarching (national) *goals and strategies for the secondary use of health data*, including the evaluation and improvement of opportunities for data access and linkage.

*“This structured, consistent thinking through while (...) properly incorporating health science expertise would be something that would be very, very important. (I-11P, pos. 56)*

They also highlighted the need to *ensure legal certainty*, including a) the harmonisation of structures, procedures and responsibilities in decision-making processes on secondary use requests and b) the establishment of appropriate mechanisms to prevent, prosecute and defend against unauthorised data sharing and reuse. Additionally, a few participants pointed to the need for measures to *ensure data security*, e.g. by continuously evaluating and adapting technical/organisational measures to the rapidly evolving opportunities for data reuse (esp. AI). Finally, *promoting trust and acceptance in health data reuse* was deemed important to increase healthcare providers’ openness to data sharing.

*“This basis of trust (…) is really quite a crucial point.” (I-13L, pos.18)*

To achieve this, emphasis should be placed on a) targeted communication strategies that convey the practical aspects of purposes, benefits and limitations of secondary use, b) the involvement of providers in the design and conduct of reuse projects, and c) the development of a constructive culture of error, the appreciation of transparency in healthcare and the improvement of providers’ knowledge about health research (“research literacy” [I-13L, pos. 26]).

## Discussion

Our exploratory qualitative study is one of the first in Germany to explore stakeholder perspectives on interests and risks that may influence healthcare providers’ willingness to share data for secondary use. The findings offer insights into how stakeholders assess risks and identify potential strategies to mitigate them. The main risks for healthcare providers reported by our participants comprise the fear of biased results and interpretation of data analyses, questionable reuse purposes, liability risks, and the disclosure of challenges (e.g. treatment errors) and misconduct (e.g. fraud) in healthcare, which may result in reputational, economic or even existential damage. While these findings align with international evidence [15–23], they also extend beyond the current literature. Particularly noteworthy here is the concern of providers that secondary analyses might reveal challenges in healthcare. This concern, which was relevant in the interviews, has received little attention in previous research. This finding is particularly striking given that transparency is at the same time seen as one of the greatest potentials of health data reuse. Furthermore, the existing literature has mainly described risks and consequences on the level of individual healthcare providers [15–21]. Our findings add risks and consequences on the meso and macro levels, such as healthcare misgovernance and loss of trust in research (see Fig. 1).

Our study also explored strategies to mitigate risks in the secondary use of health data. Strategies proposed by the interviewees include reducing the causes of risks (e.g. limitations of data sources and analytical approaches, concerns about liability or data security) by improving the (political) framework, use and access policies, and validity in secondary analyses of health data. Similar recommendations have been raised in previous studies [16–19, 23]. Our results extend previous findings by specifying how such strategies could be implemented at which levels of responsibility (e.g. policymakers, data providers and -users). Our findings also emphasise the importance of risk management in decision-making processes on secondary use requests within the respective bodies. Accordingly, risk mitigation requires transparent decision-making that involves all affected stakeholders and risk–benefit assessments that consider their various interests.

These findings point to the importance of responsibly managing the interests and risks of different stakeholder groups in decision-making about secondary use requests. Given the increasing opportunities to reuse health data for research purposes, approaches to responsible decision-making in bodies like UACs should be further developed to protect legitimate interests and mitigate potential risks without limiting the benefits of research. Normative principles for decision-making have been proposed in Daniel’s Accountability for Reasonableness framework [31] and applied to the research context [32]. According to this framework, decision making is fair if it is transparent, reasonable, revisable and regulated [31], and consistent, participatory and as free as possible from conflicts of interest [33]. Applying these principles to the key recommendations of the interviewees, we derive the following needs for action which should primarily be taken up by the stakeholders responsible for improving the underlying use and access policies (e.g. governance of initiatives like the MII, data providers) and the decision-making bodies (UACs) themselves.

### Establishing transparency about decision-making processes and results

Transparency is necessary to promote trustworthiness in decision-making [31, 34, 35]. Interviewees suggested specific strategies to improve transparency in decision-making regarding secondary use requests (e.g. making the information about the decision-making bodies, secondary use requests, and application review results public), some of which exceed the requirements of current use and access policies (e.g. MII [13]) or the recently enacted German Health Data Use Act (Gesundheitsdatennutzungsgesetz, GDNG) [36]. They therefore provide useful guidance on how to establish transparency about decision-making processes and results at a structural, procedural, and outcome level.

### Ensure reasonable and consistent decision-making

Current use and access policies do not explicitly address the risks and mitigation strategies discussed in our interviews [24], leaving their impact on secondary use request decisions unclear. Interviewees highlighted this as a critical area for improvement, which is also reflected in the principles on trustworthy decision-making and should be addressed. Reasonable and consistent decision-making requires that policies include explicit, coherent, and comprehensible criteria that enable a risk–benefit assessment that considers the interests of all affected stakeholders [31]. This would also include to clearly distinct between legitimate protection interests (e.g. against biased results) and controversial particular interests (e.g. undesired disclosure of poor quality).

### Establishing transparency about and minimising conflicts of interest, ensuring stakeholder participation

An important need for action identified in the interviews was ensuring neutral and independent use and access decisions – a key aspect also reflected in ethical perspectives on trustworthiness of decisions (e.g. [33]). Specific measures should be taken to ensure that decision-making processes a) avoid conflicts of interest or make them transparent and b) take equal account of the interests of all affected stakeholders (e.g. patients, service providers, researchers). This process should be guided by ethical principles (e.g. [34, 35, 37–39]) and involve relevant stakeholders.

### Further needs for action

Our study also highlights the need for risk mitigation strategies focused on improving the validity of secondary analyses of health data. The results point to several approaches that could help to increase confidence and trustworthiness in research. These include improving the availability and usability of data sources, as well as the infrastructure, concepts, standards and the legal framework for health data reuse (see Table 3), which interviewees believed to be the primary responsibility of data providers and policymakers. This further includes scientific efforts to evaluate and improve standards and practices to enhance quality and validity of the data used, the methodological and reporting quality, and the transparency of reuse projects.

## Limitations

While this study has some important strengths (e.g. explicit focus on mitigation strategies, variation of perspectives, deductive-inductive qualitative content analysis aiming at empirical saturation), we also acknowledge some limitations. This study was conducted between May and August 2023, a time when the draft GDNG [36], one of the most important political initiatives for digitisation, healthcare, and research in recent years in Germany, was being intensively discussed. The interviews were occasionally influenced by this discourse. Some of the strategies suggested by the interviewees are reflected in the GDNG, which has since come into force (e.g., promoting legal certainty and transparency in secondary use, improving validity, cf. Figure 1, Table 3). Still, the risk mitigation strategies discussed here offer additional insights and go beyond the GDNG.

The scope of this project was limited. Firstly, we focused on the interests of healthcare providers. Further perspectives on the topic should be complemented to develop a comprehensive framework for assessing the potentials, risks and mitigation strategies relevant to research with health data. In particular, we would like to emphasise the perspectives of patients as data donors, which were occasionally criticised by interviewees as being neglected in our study. Secondly, our study focused on the perspectives of healthcare providers in Germany. The findings therefore reflect the specific German legal, structural, and organisational context for re-using health data. However, they reflect and add to the international evidence base and are also likely to be relevant in countries with different contexts, where similar efforts are being made to make health data from different sources available for reuse (e.g. Australia, United Kingdom). Furthermore, most of the recruited stakeholders were (in-)directly linked to initiatives that aim to improve data sharing and reuse. It could therefore be assumed that the sample represented a rather positive perspective on the topic. Therefore, a focus of the snowball sampling was to recruit stakeholders with a more critical perspective and risk perception. However, we cannot determine, to what extent their perspectives were fully included. Moreover, our sampling criteria did not cover stakeholders without expertise on the topic. Given that this expertise is currently found mainly in existing initiatives focusing on university medical centres and the medical profession, our results may not adequately represent the perspectives of other healthcare providers (e.g. nurses, therapists) and the outpatient sector. However, the inclusion of varying perspectives in the sample, the data collection and analysis aimed at empirical saturation, and the findings tying up to existing evidence underline the relevance of our results in the defined scope.

## Conclusion

This exploratory qualitative study substantiates previously reported perspectives on the range of interests and risks that healthcare providers may face in the secondary use of health data. Our findings reveal risks and consequences of health data reuse that extend beyond the individual provider level to the meso- and macro levels of healthcare, as well as strategies for targeted risk mitigation. From this, we derive needs and opportunities for action that can help to reduce providers’ potential reluctance to share data and to protect their legitimate interests, without limiting the benefits of research with health data. This includes ensuring normatively reasonable decision-making processes for secondary use requests, and improving the quality, safety and trust of health data reuse. Our findings can support initiatives aiming to improve health data reuse, as well as researchers and policymakers to develop corresponding measures.

## Supplementary Information

Supplementary Material 1: Protocol deviations

Supplementary Material 2: COREQ Checklist 

Supplementary Material 3: Study team characteristics and reflexivity

Supplementary Material 4: Study material (invitation letter, study information, consent form)

Supplementary Material 5: Interview guide

Supplementary Material 6: Template Postscript

Supplementary Material 7: Final coding tree

## Data Availability

Contrary to the statement in the study information (see Suppl. 1), the data material (pseudonymized interview transcripts) will not be made publicly available due to the contextual information contained therein and the remaining risk of re-identification of the experts interviewed, most of whom hold central positions in the German healthcare system and research landscape. Access can be granted upon reasonable request to the corresponding author of the study and is subject to the consent of participants. All other instruments and documents developed and applied in this study are available as a supplement. A comprehensive German results report, which includes additional details, has been posted as a preprint (https://osf.io/vnhz9).

## Abbreviations

DIC: Data Integration Centre
EHDS: European Health Data Space
MII: Medical Informatics Initiative (Germany)
UAC: Use and Access Committee
COREQ: Consolidated criteria for reporting qualitative research
GDNG: Gesundheitsdatennutzungsgesetz (Health Data Use Act Germany)
