# Supplementary material for: Benefits and risks of health data reuse for healthcare providers: stakeholder perspectives from a qualitative interview study
Source: BMC Health Serv Res. 2025 Mar 18;25:402. doi: 10.1186/s12913-025-12500-7 (PMC11917074; doi:10.1186/s12913-025-12500-7)
Supplement: Supplementary file 1 — Supplementary Material 1: Protocol deviations [file 12913_2025_12500_MOESM1_ESM.pdf]

Supplement 1 – Deviations from the study protocol

This explorative qualitative study was preregistered (https://osf.io/uxdsa).

In section 1, “Study information” the following research questions were formulated ex-ante:

1. *What provider interests are affected by the secondary use of health data?*
2. *Can secondary use of health data be associated with challenges and risks for healthcare providers? How can these be characterized in terms of type, severity and consequences, and what relevance is attributed to them?*
3. *What would be potential mitigation strategies that could help addressing these challenges and risks, and facilitate secondary use?*

Deviation:

During data collection, the dimensional differentiation of research question 2 into “challenges” and “risks”, which was based on prior assumptions, proved to be of little help in guiding the conversations. We therefore adapted the research question by removing this distinction and focusing on risks.
