# Supplementary material for: Benefits and risks of health data reuse for healthcare providers: stakeholder perspectives from a qualitative interview study
Source: BMC Health Serv Res. 2025 Mar 18;25:402. doi: 10.1186/s12913-025-12500-7 (PMC11917074; doi:10.1186/s12913-025-12500-7)
Supplement: Supplementary file 2 — Supplementary Material 2: COREQ Checklist [file 12913_2025_12500_MOESM2_ESM.pdf]

BIH QUEST
Center for Responsible Research

Supplement 2 – COREQ Checklist

CAEHR-PI
Consolidated criteria for reporting qualitative studies (COREQ): 32-item checklist (Tong et al. 2007)

Manuscript title:

No	Item	Guide questions/description	Reported on page/line
**Domain 1: Research team and reflexivity**			
Personal Characteristics			
1.	Interviewer/facilitator	Which author/s conducted the interview or focus group?	8
2.	Credentials	What were the researcher's credentials? *E.g. PhD, MD*	Suppl. 3
3.	Occupation	What was their occupation at the time of the study?	Suppl. 3
4.	Gender	Was the researcher male or female?	Not explicitly reported, see Suppl. 3
5.	Experience and training	What experience or training did the researcher have?	Suppl. 3
Relationship with participants			
6.	Relationship established	Was a relationship established prior to study commencement?	7, Table 2<br>Suppl. 3
7.	Participant knowledge of the interviewer	What did the participants know about the researcher? e.*g. personal goals, reasons for doing the research*	Suppl. 3, 4a-c
8.	Interviewer characteristics	What characteristics were reported about the interviewer/facilitator? e.g. *Bias, assumptions, reasons and interests in the research topic*	Suppl. 3
**Domain 2: study design**			
Theoretical framework			
9.	Methodological orientation and Theory	What methodological orientation was stated to underpin the study? *e.g. grounded theory, discourse analysis, ethnography, phenomenology, content analysis*	6
Participant selection			
10.	Sampling	How were participants selected? *e.g. purposive, convenience, consecutive, snowball*	6f
11.	Method of approach	How were participants approached? e.*g. face-to-face, telephone, mail, email*	7
12.	Sample size	How many participants were in the study?	9
13.	Non-participation	How many people refused to participate or dropped out? Reasons?	9
Setting			
14.	Setting of data collection	Where was the data collected? e.*g. home, clinic, workplace*	8
15.	Presence of non-participants	Was anyone else present besides the participants and researchers?	8
16.	Description of sample	What are the important characteristics of the sample? *e.g. demographic data, date*	9f, Table 1
Data collection			
17.	Interview guide	Were questions, prompts, guides provided by the authors? Was it pilot tested?	8, Table 1, Suppl. 5
18.	Repeat interviews	Were repeat interviews carried out? If yes, how many?	8
19.	Audio/visual recording	Did the research use audio or visual recording to collect the data?	8
20.	Field notes	Were field notes made during and/or after the interview or focus group?	8, Suppl. 6
21.	Duration	What was the duration of the interviews or focus group?	Table 2
22.	Data saturation	Was data saturation discussed?	7

23.	Transcripts returned	Were transcripts returned to participants for comment and/or correction?	No
**Domain 3: analysis and findings**			
Data analysis			
24.	Number of data coders	How many data coders coded the data?	9
25.	Description of the coding tree	Did authors provide a description of the coding tree?	10; Figure 1, Supplement 6
26.	Derivation of themes	Were themes identified in advance or derived from the data?	9
27.	Software	What software, if applicable, was used to manage the data?	9
28.	Participant checking	Did participants provide feedback on the findings?	8
**Reporting**			
29.	Quotations presented	Were participant quotations presented to illustrate the themes / findings? Was each quotation identified? e.*g. participant number*	11-20
30.	Data and findings consistent	Was there consistency between the data presented and the findings?	
31.	Clarity of major themes	Were major themes clearly presented in the findings?	11-20, Figure 1
32.	Clarity of minor themes	Is there a description of diverse cases or discussion of minor themes?	11, 12, 15, 18, 19

Reference:

Tong A, Sainsbury P, Craig J. Consolidated criteria for reporting qualitative research (COREQ): a 32-item checklist for interviews and focus groups. International Journal for Quality in Health Care. 2007. Volume 19, Number 6: pp. 349 – 357
