# Supplementary material for: Benefits and risks of health data reuse for healthcare providers: stakeholder perspectives from a qualitative interview study
Source: BMC Health Serv Res. 2025 Mar 18;25:402. doi: 10.1186/s12913-025-12500-7 (PMC11917074; doi:10.1186/s12913-025-12500-7)
Supplement: Supplementary file 3 — Supplementary Material 3: Study team characteristics and reflexivity [file 12913_2025_12500_MOESM3_ESM.docx]

**Supplement 3 – Study team characteristics & positioning**

The interdisciplinary study team consisted of a physician/bioethicist (DS, Prof.), a health scientist (ST, Dr.), a pharmacist (SGS, PhD) and a clinical psychologist (MMP, PhD). All members of the study team are part of the working group (WG) “Translational Bioethics and Meta-Research” at the QUEST Center for Responsible Research of the Berlin Institute of Health at Charité - Universitätsmedizin Berlin, which is led by DS. This WG aims to improve the trustworthiness, usefulness and ethics of biomedical research by analysing challenges and developing solutions. The team members bring different professional backgrounds to their scientific work, including medical ethics (DS), health services research (ST), medical guideline development and advocacy (SGS) and clinical psychology (MMP). In addition, DS (physician) and ST (occupational therapist) have been involved in the clinical care of patients in different settings in the past. The reuse of health and other data is subject of research of all team members, but they do not currently use this data themselves for research purposes. The different knowledge and experiences of the team members were incorporated into the joint reflection on the study design, data collection and analysis. This increased both the diversity of perspectives while reflecting on the data, the material and the emerging findings, as well as the requirements for reducing complexity – particularly while developing a common understanding during data analysis and results reporting. Following a systematic literature search on the topic, the team members had prior knowledge of the type and content of the available evidence. This knowledge was consciously incorporated as preliminary assumptions into the development of the instruments for data collection and analysis, while recognising its preliminary nature. For example, preliminary assumptions informed the development of follow-up questions (e.g. expectations about the dimensionality of an aspect), but did not shape the structure or main content of the interview guide. In turn, the deductively defined main categories of the coding tree were based on the core dimensions of the interview guide and were further developed inductively during data analysis.

All team members are experienced in qualitative research. None of the team members had any contact with the participants prior to the study, with the exception of DS, who knew few of the participants through his professional network and his association with the Medical Informatics Initiative (MII) as spokesperson of the WG “Consent”. Entry into the field and relationships with the participants were therefore primarily characterised by an external researcher perspective without direct relation to the secondary use of health data. Data collection and analysis were led by the first author (ST), who has several years of experience in qualitative research and teaching in qualitative research methods. It became apparent through explicit statements in the interviews that some participants, prompted by our interview questions, gained new perspectives on the topic. We interpreted this to mean that both the research questions and the interview questions led to a sensitisation – and thus to an influence – in the field, which was expressed in the discursive and reflexive discussion of the topic during the interviews. The extent to which this influence is positive or negative could, in our view, best be assessed prospectively via the further uptake of the topic in the field. A positive influence could, for example, be an intensified and differentiated discourse on risks and their management, while a negative influence could be an increased reluctance towards the secondary use of health data.
