# Supplementary material for: Benefits and risks of health data reuse for healthcare providers: stakeholder perspectives from a qualitative interview study
Source: BMC Health Serv Res. 2025 Mar 18;25:402. doi: 10.1186/s12913-025-12500-7 (PMC11917074; doi:10.1186/s12913-025-12500-7)
Supplement: Supplementary file 4 — Supplementary Material 4: Study material (invitation letter, study information, consent form) [file 12913_2025_12500_MOESM4_ESM.zip › CAEHR_PI_Suppl4a_Template_Invitation.pdf]

Supplement 4a – Template invitation interview (English translation)

Dear ...,

We would like to invite you to take part in an interview.

In the interview study "**Provider Interests in Secondary Use of Health Data**" at the QUEST Center for Responsible Research at the Berlin Institute of Health (BIH), we are investigating potential challenges and risks that healthcare providers (e.g. hospitals) may face when making health data available for secondary use.
In contrast to other fundamental aspects of the secondary use of health data (e.g. data protection and security), this topic has so far hardly been addressed. However, perceived challenges and risks may lead to reluctance towards the provision, processing and analysis of secondary data.

This study is being conducted in the context of the digital hub CAEHR, which is part of the Medical Informatics Initiative funded by the BMBF.
Your perspective on this topic as a [personalised] is of great importance for this study.
You can contribute to establish a better understanding of this aspect of the secondary use of health data for future research and political decisions.

The **interview** will focus on the following **key topics**:

- Interests of healthcare providers in the context of secondary use of health data
- Challenges and risks for healthcare providers associated with the secondary use of health data, risk characteristics
- Strategies for addressing challenges and risks

Please find background information on the study and comprehensive information on confidentiality and data protection in the attached study information and the study protocol.
We would like to ask you to contact us for scheduling an interview. We will take your needs into account when making an appointment.
The interview will last approximately **40 to 60 minutes** an can be conducted **online or in-person**. We offer a compensation of €150 for your time and effort.

If you agree to participate in our study, please fill in the attached consent form and return it to us by e-mail. We would also be grateful if you could provide us with the contact details of any colleagues who may be interested in participating in this study.

Please contact me, Susanne Stark, if you have any questions.
Kind regards,

BIH QUEST
Center for Responsible Research

Supplement 4a – Vorlage Einladung Interview (German original)

Sehr geehrte/r ...,

wir möchten Sie einladen, an einem Interview teilzunehmen.

In der Interviewstudie „**Provider Interests in Secondary Use of Health Data**" am QUEST Center for Responsible Research des Berlin Institute of Health (BIH) befassen wir uns mit möglichen Herausforderungen und Risiken, die für Leistungserbringer (z. B. Krankenhäuser) entstehen können, wenn sie Gesundheitsdaten für die Sekundärnutzung zur Verfügung stellen.
Dieses Thema wurde, anders als andere grundlegende Fragestellungen zur Sekundärnutzung von Gesundheitsdaten (z.B. Datenschutz und -sicherheit), bislang noch kaum beleuchtet. Allerdings können wahrgenommene Herausforderungen und Risiken zu Vorbehalten gegenüber der Bereitstellung, Aufbereitung und Analyse von Sekundärdaten führen.

Diese Studie wird im Kontext des Digitalen Fortschrittshubs CAEHR als Teil der vom BMBF geförderten Medizininformatik-Initiative (MII) durchgeführt.
Ihrer Perspektive auf dieses Thema als [personalisiert] kommt in dieser Studie hohe Bedeutung bei. Sie können in einem Interview dazu beitragen, diesen Aspekt der Sekundärnutzung von Gesundheitsdaten für künftige Forschungsarbeiten und politische Entscheidungen näher zu beleuchten.

Das **Interview** wird sich auf die folgenden **Kernthemen** konzentrieren:

- Interessen von Leistungserbringern im Kontext der Sekundärnutzung von Gesundheitsdaten
- Herausforderungen und Risiken für Leistungserbringer in Verbindung mit der Sekundärnutzung von Gesundheitsdaten, Merkmale von Risiken
- Strategien, um Herausforderungen und Risiken zu adressieren

Weitere Hintergrundinformationen zur Studie sowie ausführliche Informationen zu Vertraulichkeit und Datenschutz entnehmen Sie bitte der beigefügten Studieninformation und dem Studienprotokoll.
Wir möchten Sie bitten, Kontakt mit uns aufzunehmen für die Vereinbarung eines Interviewtermins. Für die Terminfindung orientieren wir uns natürlich an Ihrem Kalender. Das Interview kann **online oder persönlich** bei Ihnen vor Ort durchgeführt werden und umfasst eine voraussichtliche Dauer von etwa **40-60 Minuten**. Wir bieten Ihnen eine Aufwandsentschädigung von 150 € für Ihre Zeit und Mühe.

Wenn Sie bereit sind, an der Studie teilzunehmen, füllen Sie bitte die beigefügte Einverständniserklärung aus und senden Sie das Dokument per E-Mail an uns zurück.
Wenn Sie zudem weitere geeignete Kolleg*innen kennen, die Interesse an der Teilnahme an dieser Studie haben könnten, würden wir uns freuen, wenn Sie uns deren Namen mitteilen.

Für weitere Fragen stehe ich, Susanne Stark, Ihnen jederzeit gern zur Verfügung.
Mit freundlichen Grüßen,
