# Supplementary material for: Benefits and risks of health data reuse for healthcare providers: stakeholder perspectives from a qualitative interview study
Source: BMC Health Serv Res. 2025 Mar 18;25:402. doi: 10.1186/s12913-025-12500-7 (PMC11917074; doi:10.1186/s12913-025-12500-7)
Supplement: Supplementary file 4 — Supplementary Material 4: Study material (invitation letter, study information, consent form) [file 12913_2025_12500_MOESM4_ESM.zip › CAEHR_PI_Suppl4b_Study_information.pdf]

Supplement 4b – Study Information (English translation)

Study Information

PROJECT: Provider Interests in Secondary Use of Health Data – Stakeholder Interviews

Dear prospective participant,

We hereby offer you the opportunity to participate in a scientific interview study, which is being conducted at the QUEST Center for Responsible Research at the Berlin Institute of Health (BIH). This interview study focusses the investigation of interests and, in particular, risks healthcare providers may face in the context of the secondary use of health data.
With this document we will inform you about the background and objectives of this study. Furthermore, we wish to let you know for which purposes and how we will capture, process and store your personal data. In addition, you will find information on how to exercise your rights as a data subject. Your data may only be processed with your consent, which we hereby ask you to give. Please read the following information carefully and contact us should you have any further questions (see below for contact details).

Purpose of data collection and processing

- Our study investigates the range of potential interests and, in particular, risks to healthcare providers (e.g. hospitals) that may be associated with third parties accessing, processing and analysing health data for research purposes.
- We are conducting an interview study with you and a small number of other stakeholders to better understand these potential risks to healthcare providers associated with making health data available for secondary use. In particular, we aim to understand what characterises potential risks and their significance. We also want to explore the extent to which the nature and context of secondary use projects influence these risks and what strategies can help to mitigate and manage them.

1. Responsibility

The BIH QUEST Center is responsible for the conduct of this research project and data processing in accordance with ethical codes of conduct and data privacy legislation. For BIH QUEST Center contact details please see Section 6 of this document. This project titled “Provider Interests in Secondary Use of Health Data” received a positive vote from the Ethics Committee of Charité – Universitätsmedizin Berlin. The data protection concept for the project, including data protection declaration and consent form, was reviewed and approved by Charité’s Data Protection and Governance department.

2. Information on data processing

Interviewees are contacted via email, using a publicly available email address or the email address that was shared with the researchers after a contact person asked the potential interviewee whether they agreed to share their email address. This information will be stored on a Charité server in such a way that it is inaccessible to third parties and separate from interview-related data. The information will be protected by access restrictions and an access authorization system. The information provided by you during the interview will be captured as interview-related data.
The interviews will be recorded electronically as audio files. These audio files, which include your voice as an identifying feature, will be pseudonymized and transcribed as text by a contractor so that they may be analysed using qualitative text analysis methods. The identities of the participants as well as names of other persons and personally identifiable information will be redacted from these transcripts by deleting or altering the

corresponding passages and identifiers. In this way, all interview transcripts will be pseudonymized. We do not intend to use the data in the transcripts to reconstruct any personal identities. However, we cannot rule out the possibility that the information provided may be linked to specific persons in individual cases.
All data will be processed on Charité servers by employees of the BIH QUEST Center, with the exception of the audio file transcriptions, which will be performed by the contractor.

Duration of data storage and criteria for determining this duration

Interview audio files will be deleted once the transcriptions have been checked. Your contact details (email address) will be stored on Charité servers until the end of the project. Once the project is completed, these data will be deleted. Your interview-related data – in this case, the pseudonymized interview transcripts – will be stored for at least 10 years in accordance with Charité's Good Scientific Practice statutes. Storage beyond this time frame is sought for two reasons: (i) for the purpose of long-term re-use (ii) to allow for long-term comparisons.

Sharing data (with recipient/third party) and publication

Interview audio files will be sent to a contractor for the purpose of transcription. The contractor will sign a confidentiality obligation. We intend to publish the results of the project in a form that will not reveal your identity nor the identity of any other person or institution that you mention in the interview. With your consent, we plan to make the pseudonymized interview transcripts accessible for the purposes of transparency and subsequent use in research. The interview transcripts will not relate to identifiable natural persons. You will receive a copy of the pseudonymized transcript, with the option to revise or edit if any privacy concerns arise. When we publish our findings, we plan to deposit the transcripts in an appropriate repository, e.g. the Repository of the Research Data Centre for Higher Education Research and Science Studies (des Forschungsdatenzentrums für Hochschulforschung und Wissenschaftsforschung, FDZ) https://www.fdz.dzhw.eu/de.

3. Benefits and risks

Besides the small risk of re-identification, there are no risks associated with taking part in the interview. Participants are free to decline responding to one or more questions. We will exploit all technological possibilities to protect your privacy. This includes our contractual guarantee to use the interview solely for the stated purposes, as well as the contractual guarantee of confidentiality from our collaborators and contractors.

4. Legal basis for data processing

The legal basis for our processing of your data is your consent (Art. 6 para. 2 lit. a GDPR). You may withdraw your consent at any time without providing a reason and object to the processing of the information provided by you. This withdrawal will only apply prospectively.

5. Cancellation policy, data subject rights, contacting us

Your participation in the interview is voluntary. You may choose to discontinue your participation at any time. Your rights as a data subject regarding the personal data processed in the context of this project are as follows:

(1) You may withdraw your given consent at any time; your withdrawal will be effective for the future (Art. 7 GDPR). The lawfulness of data processing carried out prior to the withdrawal will remain unaffected.
(2) You have the right to be informed about the personal data stored about you. You also have the right to rectification and deletion of your data; the right to restriction of processing of your data; and the right to data portability. **Please note** that once your contact details have been deleted, no withdrawal of consent, and no information, correction, deletion or blocking of your data will be possible, as we will no longer be able to assign the data to your person.

To exercise the aforementioned rights, please contact the organisational unit responsible for processing personal data: Charité - Universitätsmedizin Berlin, Charitéplatz 1, 10117 Berlin. Executing unit: BIH QUEST Center for Responsible Research, Berlin Institute of Health at Charité, Anna-Louisa-Karsch-Strasse 2, 10178 Berlin. Contact details: Daniel Strech, Phone: +49 30 450 543 068, E-Mail: daniel.strech@bih-charite.de For questions

regarding data processing and compliance with privacy protection requirements, you may also contact the Charité data protection officer: Data Protection Officer of Charité - Universitätsmedizin Berlin, Charitéplatz 1, 10117 Berlin, email: datenschutzbeauftragte@charite.de. If you suspect illegal data processing, you may file a complaint with the supervisory authority responsible for Charité – Universitätsmedizin Berlin. Please direct your informal complaint to: Berlin Commissioner for Data Protection and Freedom of Information, Friedrichstraße 219, 10969 Berlin, email: mailbox@datenschutz-berlin.de, phone: +49 30 13889-0.

6. Costs and compensation

There are no costs involved in your interview participation. You will receive a honorarium of 150€ for your participation. Typically, the interviewer(s) will visit the interviewees or the interviews will be conducted online.

Supplement 4b – Study Information (German original)

Studieninformation

Version 3.0 | 2023-03-27

PROJEKT: Provider Interests in Secondary Use of Health Data – Stakeholder Interviews

Sehr geehrte*r Studieninteressent*in,

hiermit bieten wir Ihnen die Teilnahme an einer wissenschaftlichen Interviewstudie (Befragung) an, die am QUEST Center for Responsible Research des Berlin Institute of Health (BIH) durchgeführt wird. Im Zentrum dieser Interviewstudie steht die Untersuchung von Interessen und insbesondere Risiken für Leistungserbringer, die im Zuge der Sekundärnutzung von Gesundheitsdaten entstehen können. Mit diesem Dokument informieren wir Sie über den Hintergrund und die Ziele der Studie. Darüber hinaus informieren wir Sie darüber, zu welchen Zwecken bzw. auf welche Art und Weise Ihre Informationen erfasst, verarbeitet und gespeichert werden. Zudem finden Sie Informationen dazu, wie Sie Ihre Betroffenenrechte wahrnehmen können. Die Verarbeitung Ihrer Daten erfolgt auf der Grundlage Ihrer Einwilligung, um die wir Sie hiermit bitten. Bitte lesen Sie diese sorgfältig durch und wenden Sie sich gern an uns, wenn Sie weitere Fragen haben (Kontaktdaten, siehe unten).

Zweck der Datenerfassung und –Verarbeitung (Studieninformation)

- Unsere Studie untersucht das Spektrum möglicher Interessen und vor allem wahrgenommener Risiken, die für Leistungserbringer (z. B. Krankenhäuser) in Verbindung mit dem Zugang zu, dem Zugriff auf sowie der Aufbereitung und Analyse von Sekundärdaten durch Dritte einhergehen können.
- Wir führen eine Interviewstudie mit Ihnen und einer kleinen Anzahl Stakeholder durch, um diese Risiken für Leistungserbringer besser zu verstehen. Insbesondere wollen wir verstehen, was mögliche Risiken charakterisiert und welche Bedeutung ihnen in der Sekundärnutzung von Gesundheitsdaten zukommt. Darüber hinaus wollen wir untersuchen, inwieweit die Art und der Kontext der Sekundärdatennutzung diese Risiken beeinflussen und welche Strategien zu ihrer Minimierung und zu ihrem Management beitragen können.

1. Verantwortliche

Verantwortlich für die Durchführung und Datenverarbeitung dieses Forschungsprojekts im Einklang mit ethischen Verhaltenskodizes und Datenschutzgesetzen ist das BIH QUEST Center. Die Kontaktdaten des BIH QUEST Centers sind unter Abschnitt 6 zu finden. Das Projekt mit dem Titel "Provider Interests in the Secondary Use of Health Data " erhielt ein positives Votum der Ethikkommission der Charité - Universitätsmedizin Berlin. Das Datenschutzkonzept zum Vorhaben, einschließlich der Datenschutzerklärung und Einwilligungserklärung, wurden durch den Geschäftsbereich Datenschutz und Governance der Charité begutachtet und freigegeben.

2. Informationen zur Datenverarbeitung

Interviewpartner*innen werden per E-Mail kontaktiert. Dafür wird eine öffentlich zugängliche E-Mail-Adresse oder die E-Mail-Adresse verwendet, die den Forscher*innen mitgeteilt wurde, nachdem eine Kontaktperson den/die mögliche Interviewpartner*in gefragt hat, ob er mit der Weitergabe seiner bzw. ihrer E-Mail-Adresse einverstanden ist. Diese Angaben werden für Dritte unzugänglich und separat von erhebungsbezogenen Daten auf

einem Charité-Server aufbewahrt bzw. gespeichert. Sie sind durch Zugangsbeschränkungen und Zugriffsberechtigungen geschützt. Die von Ihnen während des Interviews gemachten Angaben werden als befragungsbezogene Daten erfasst.
Die Interviews werden elektronisch in Form von Audiodateien aufgezeichnet. Diese Audiodateien, auf denen Ihre Stimme als identifizierendes Merkmal gespeichert ist, werden von einem beauftragten Unternehmen zeitnah pseudonymisiert und in einen Text transkribiert, um sie mithilfe qualitativer textanalytischer Verfahren analysieren zu können. In den Interviewtranskripten werden die Identität der Interviewpartner*innen sowie die Namen anderer Personen sowie personenidentifizierbarer Informationen durch Löschung bzw. Verfremdung bereinigt und die Interviewtranskripte auf diese Weise pseudonymisiert. Wir beabsichtigen nicht, die Daten in den Transkripten zu verwenden, um persönliche Identitäten zu rekonstruieren. Es kann jedoch im Einzelfall nicht ausgeschlossen werden, dass die Angaben im Einzelfall mit bestimmten Personen in Verbindung gebracht werden können.
Die Datenverarbeitung geschieht auf Servern der Charité durch Mitarbeiter*innen des BIH QUEST Centers mit Ausnahme der Transkription der Audioaufzeichnungen aus den Interviews, diese erfolgt beim beauftragten Unternehmen.

Dauer der Speicherung der Daten bzw. Kriterien der Festlegung dieser Dauer

Die Audiodateien der Interviews werden nach Prüfung der Transkription gelöscht. Ihre Kontaktdaten (E-Mail-Adresse), werden bis zum Projektende auf Charité-Server gespeichert und anschließend gelöscht. Ihre interviewbezogenen Daten – in diesem Fall die pseudonymisierten Interviewtranskripte - werden im Einklang mit der Satzung Gute Wissenschaftliche Praxis der Charité für mindestens 10 Jahre gespeichert. Eine Speicherung über diesen Zeitpunkt hinaus wird aus zwei Gründen angestrebt: (i) zum Zweck der langfristigen Nachnutzung (ii) um Langzeitvergleiche anstellen zu können.

Weiterleitung (Empfänger/Dritte) und Veröffentlichung

Die Audiodateien werden zum Zwecke der Transkription an ein beauftragtes Unternehmen übersandt. Das beauftragte Unternehmen wird eine Vertraulichkeitserklärung unterzeichnen. Wir beabsichtigen, die Ergebnisse des Projekts in einer Form zu veröffentlichen, die weder Ihre Identität noch die Identität einer anderen Person oder Institution, die Sie im Interview erwähnen, preisgibt. Mit Ihrem Einverständnis planen wir, die pseudonymisierten Interviewprotokolle zum Zwecke der Transparenz und der späteren Verwendung in der Forschung zugänglich zu machen. Die Interviewtranskripte werden sich nicht auf identifizierbare natürliche Personen beziehen. Sie erhalten eine Kopie des pseudonymisierten Transkripts mit der Möglichkeit, es zu überarbeiten, falls Bedenken hinsichtlich des Datenschutzes bestehen. Wenn wir unsere Ergebnisse veröffentlichen, planen wir, die Transkripte in einem geeigneten Repositorium zu hinterlegen, z.B. im Repositorium des Forschungsdatenzentrums für Hochschulforschung und Wissenschaftsforschung (FDZ) https://www.fdz.dzhw.eu/de.

3. Nutzen und Risiken

Abgesehen von dem geringen Risiko der Re-Identifizierung sind keine Risiken vorhanden. Es steht den Teilnehmer*innen frei, Die Beantwortung einer oder mehrerer Fragen abzulehnen. Wir werden alle technischen Möglichkeiten ausschöpfen, um Ihre Privatsphäre zu schützen. Dazu gehört unsere vertragliche Zusicherung, dass wir das Interview ausschließlich zu den angegebenen Zwecken verwenden werden sowie die vertragliche Zusicherung der Vertraulichkeit unserer Kooperationspartner und Auftragnehmer.

4. Rechtsgrundlage der Datenverarbeitung

Rechtsgrundlage für die Verarbeitung Ihrer Daten durch uns ist Ihre Einwilligung (Art. 6 Abs. 2 lit. a DSGVO). Sie können Ihre Einwilligung jederzeit ohne Angaben von Gründen widerrufen und einer Weiterverarbeitung Ihrer Daten widersprechen. Dieser Widerruf wirkt sich nur für die Zukunft aus.

5. Widerrufsbelehrung, Rechte der Betroffenen und Kontaktaufnahme

Ihre Teilnahme an dem Interview ist freiwillig. Sie können jederzeit von Ihrer Teilnahme zurücktreten. *Sie haben im Zusammenhang mit den im Rahmen dieses Projekts verarbeiteten personenbezogen Daten folgende Rechte:*

(1) Sie können Ihre erteilte Einwilligung jederzeit mit Wirkung auf die Zukunft widerrufen (Art. 7 DSGVO). Die Rechtmäßigkeit der bis zum Widerruf erfolgten Datenverarbeitung bleibt davon unberührt.
(2) Sie haben das Recht auf Auskunft über die zu Ihrer Person gespeicherten Daten. Zudem haben Sie das Recht auf Berichtigung und Löschung Ihrer Daten, das Recht auf Einschränkung der Verarbeitung sowie das Recht auf Datenübertragbarkeit. **Bitte beachten Sie**, dass ab dem Zeitpunkt der Löschung Ihrer Kontaktdaten kein Widerruf der Einwilligung, keine Auskunft über oder Berichtigung, Löschung bzw. Sperrung Ihrer Daten mehr möglich ist, da wir die Daten nicht mehr Ihrer Person zuordnen können.

Zur Wahrnehmung der vorgenannten Rechte wenden Sie sich bitte an die für die Verarbeitung der personenbezogenen Daten verantwortliche Stelle: Charité – Universitätsmedizin Berlin, Körperschaft des öffentlichen Rechts, Chariteplatz 1, 10117 Berlin. Ausführende Stelle: BIH QUEST Center for Responsible Research, Berlin Institute of Health at Charité, Anna-Louisa-Karsch-Straße 2, 10178 Berlin. Kontakt: Daniel Strech, Tel.: +49 30 450 543 068, E-Mail: daniel.strech@bih-charite.de. Bei Fragen zur Datenverarbeitung und zur Einhaltung der datenschutzrechtlichen Anforderungen können Sie sich auch an die Datenschutzbeauftragte der Charité wenden: Behördliche Datenschutzbeauftragte der Charité – Universitätsmedizin Berlin, Chariteplatz 1, 10117 Berlin, E-Mail: datenschutzbeauftragte@charite.de. Für den Fall, dass Sie eine Datenverarbeitung für rechtswidrig halten, haben Sie die Möglichkeit, bei der für die Charité - Universitätsmedizin Berlin zuständigen Aufsichtsbehörde Beschwerde einzureichen. Die Beschwerde kann formlos erfolgen: Berliner Beauftragte für Datenschutz und Informationsfreiheit, Friedrichstraße 219, 10969 Berlin, E-Mail: mailbox@datenschutz-berlin.de, Tel.: +49 30 13889-0.

6. Kosten und/oder Entschädigungen

Mit Ihrer Interviewteilnahme sind keine Kosten verbunden. Sie erhalten ein Honorar von 150€ für Ihre Teilnahme. In der Regel besuchen die Interviewer die Befragten oder die Interviews werden online durchgeführt.
