# Supplementary material for: Benefits and risks of health data reuse for healthcare providers: stakeholder perspectives from a qualitative interview study
Source: BMC Health Serv Res. 2025 Mar 18;25:402. doi: 10.1186/s12913-025-12500-7 (PMC11917074; doi:10.1186/s12913-025-12500-7)
Supplement: Supplementary file 4 — Supplementary Material 4: Study material (invitation letter, study information, consent form) [file 12913_2025_12500_MOESM4_ESM.zip › CAEHR_PI_Suppl4c_Consent_form.pdf]

Supplement 4c – Consent form (English translation)

Informed Consent
PROJECT: Provider Interests in Secondary Use of Health Data – Stakeholder Interviews

This is a research project conducted by the QUEST Center for Responsible Research at the Berlin Institute of Health (BIH). You are invited to participate in this research project by taking part in an interview. Interviews are conducted under the authority of the QUEST Center for Responsible Research, Berlin Institute of Health (BIH) at Charité – Universitätsmedizin Berlin, Anna-Louisa-Karsch-Str. 2, D-10187 Berlin.

Project lead:
Susanne Stark & Prof. Dr. Dr. Daniel Strech

Interviewee: [name, e-mail]
Interviewer: [name]

I hereby declare that
- I have received and read the study information of the project "Provider Interests in the Secondary Use of Health Data – Stakeholder Interviews", version 3.0 dated 27.03.2023.
- I have received written information on the nature, significance, and risks involved in research relating to the aforementioned project.
- I have had sufficient opportunity to settle all remaining questions with the person stated above.

I have been informed
- that I may withdraw my consent to future data processing at any time and that the withdrawal of my consent will not affect the lawfulness of data processing that may have been conducted prior to my withdrawal of consent. My withdrawal of consent will have no consequences for me, even if this would significantly hinder or prevent the implementation of the research project.

I am aware
- of my rights to information, correction, and deletion of my personal data captured in the context of this study. If I wish to exercise these rights, I will approach the project lead of the study, who will be able to identify my personal data. Furthermore, I may file a complaint with the relevant data protection authority.
- that from the time of deletion of my contact data, no withdrawal of consent, information, correction, deletion or blocking of my data will be possible as the data can no longer be assigned to my person.
- my personal data required for the aforementioned project as identified in the Study Information dated 27.03.2023 may be captured, recorded, pseudonymized, and processed, including on electronic media.
- that results of the study may be published in pseudonymized form that cannot be linked to my person.
- that the leads of this study or their contractors may contact me in order to obtain clearance of my transcript in the event of publication.

I have received copies of the aforementioned Study Information and the Declaration of Consent.

______________________________	______________________________
Place, Date	Signature interviewee

I hereby declare consent to the collection and processing of my personal data by the study team for the purpose of conducting the study and agree to participate in the interview. My consent is given voluntarily.

I hereby declare consent:

______________________________ ______________________________

Place, Date Signature interviewee

I hereby declare that I have informed the participant in writing about the nature, significance, and risks of the aforementioned project and handed him/her copies of the Study Information and the Declaration of Consent. The verbal and written information was provided on [please insert date].

______________________________ ______________________________

Place, Date Signature interviewer

Supplement 4c – Consent form (German original)

Einwilligungserklärung

Version 3.0 | 2023-03-27

PROJEKT: Provider Interests in Secondary Use of Health Data – Stakeholder Interviews

Dies ist ein Forschungsprojekt des BIH QUEST Center for Responsible Research des Berlin Institute of Health (BIH). Sie sind eingeladen, sich an diesem Forschungsprojekt zu beteiligen, indem Sie an einem Interview teilnehmen. Verantwortlich für die Durchführung der Interviews ist das QUEST Center for Responsible Research, Berlin Institute of Health (BIH) at Charité – Universitätsmedizin Berlin, Anna-Louisa-Karsch-Str. 2, D-10187 Berlin.

Projektleitung:
Susanne Stark & Prof. Dr. Dr. Daniel Strech

Interviewte Person: [Name, e-Mail]
Interviewer*in: [Name]

Hiermit erkläre ich, dass
- ich die Studieninformation zum Projekt „Provider Interests in the Secondary Use of Health Data – Stakeholder Interviews" in der Version 3.0 vom 27.03.2023 erhalten und gelesen habe.
- ich schriftlich über das Wesen, die Bedeutung und die Risiken der der Forschung im Zusammenhang mit dem o.g. Projekt informiert wurde.
- ich ausreichend Gelegenheit hatte, alle offenen Fragen mit den oben genannten Personen zu klären.

Ich wurde darüber aufgeklärt,
- dass ich meine Einwilligung in die Datenverarbeitung jederzeit für die Zukunft widerrufen kann und dass der Widerruf die Rechtmäßigkeit der Datenverarbeitung, die möglicherweise vor meinem Widerruf der Einwilligung erfolgt ist, nicht berührt. Mein Widerruf der Einwilligung hat keine Auswirkungen für mich, auch wenn hierdurch die Durchführung des Forschungsvorhabens erheblich beeinträchtigt oder verhindert wird.

Mir ist bewusst, dass
- ich über Rechte auf Auskunft, Berichtigung und Löschung meiner im Rahmen dieser Studie erhobenen personenbezogenen Daten verfüge. Wenn ich diese Rechte ausüben möchte, wende ich mich an die Projektleitung der Studie, die in der Lage sein wird, meine personenbezogenen Daten zu identifizieren. Außerdem kann ich eine Beschwerde bei der zuständigen Datenschutzbehörde einreichen.
- ab dem Zeitpunkt der Löschung meiner Kontaktdaten kein Widerruf der Einwilligung, Auskunft, Berichtigung, Löschung oder Sperrung meiner Daten mehr möglich ist, da die Daten nicht mehr meiner Person zugeordnet werden können.
- meine in der Studieninformation vom 27.03.2023 genannten, für das o.g. Projekt erforderlichen personenbezogenen Daten erfasst, gespeichert, pseudonymisiert und verarbeitet werden dürfen, auch auf elektronischen Medien.
- Ergebnisse der Studie in pseudonymisierter Form, die nicht mit meiner Person in Verbindung gebracht werden können, veröffentlicht werden dürfen.
- dass die Leitung dieser Studie oder ihre Auftragnehmer mit mir Kontakt aufnehmen dürfen, um im Falle einer Veröffentlichung die Freigabe meiner Abschrift zu erhalten.

Ich habe eine Kopie der oben genannten Studieninformation und der Einverständniserklärung erhalten

Ort, Datum	Unterschrift Teilnehmer*in
______________________	______________________

Ich erkläre mich mit der Erhebung und Verarbeitung meiner personenbezogenen Daten durch das Studienteam zum Zweck der Durchführung der Studie einverstanden und willige in die Teilnahme an einem Interview ein. Meine Zustimmung ist freiwillig.

Ich erkläre hiermit mein Einverständnis:

Ort, Datum	Unterschrift Teilnehmer*in

Hiermit erkläre ich, dass ich den/die Teilnehmer*in schriftlich über Art, Bedeutung und Risiken des oben genannten Projekts aufgeklärt und ihm/ihr Kopien der Studieninformation und der Einverständniserklärung ausgehändigt habe. Die mündliche und schriftliche Aufklärung erfolgte am [bitte Datum einfügen].

Ort, Datum	Unterschrift Interviewer*in
