# Supplementary material for: Benefits and risks of health data reuse for healthcare providers: stakeholder perspectives from a qualitative interview study
Source: BMC Health Serv Res. 2025 Mar 18;25:402. doi: 10.1186/s12913-025-12500-7 (PMC11917074; doi:10.1186/s12913-025-12500-7)
Supplement: Supplementary file 5 — Supplementary Material 5: Interview guide [file 12913_2025_12500_MOESM5_ESM.pdf]

Supplement 5 – Interview guide (English translation)

Study: **Provider Interests in the Secondary Use of Health Data**
Interview guide – Stakeholder interviews

Introduction

Introduction of interviewers & project (see background information)

Questions?

Check **consent form &** inform about **recording**

Part 1: Intro – Introduction & opening question

1. Could you please describe the focus of your work and to what extent the secondary use of health data plays a role in it?
    - Against this background, what experiences did you make in the secondary use of health data?
2. In general: From your perspective, what interests of service providers are affected by the secondary use of health data??
    - What interests could promote/impede this kind of secondary use?

Part 2: Secondary use: Risks, their characteristics, consequences & influences

1. What is your assessment: Can secondary use of health data be associated with risks for healthcare providers?
    - Can you please explain your assessment in more detail / What leads you to the conclusion that secondary use poses (no/low/high) risks?

2. From your perspective, what are the specific risks for healthcare providers associated with the secondary use of health data?
    - At what points of secondary use do these risks arise and how?

Refers to: Data request/review, data access, data processing/analysis, publication

    - To what extent do these risks differ depending on the type of healthcare provider involved (e.g. inpatient vs. outpatient)?

3. To what extent do the type and context of secondary use projects/research questions influence the risks you have mentioned?

If necessary, provide information: Potential risks based on previous discussion in research group/literature review: **Type of data and data access** (e.g. anonymised/pseudonymised; aggregated vs. individual data) **research objectives** (e.g. bearing risk, similar projects planned within own institution) **Person/Party** requesting data (legal status [e.g. research institution/for-profit private company], reputation, origin, other parties involved)

    - Can you please explain [mentioned characteristics] in more detail? What factors specifically constitute the risk of such projects/research questions?

4. What negative consequences could result if such risks are not adequately addressed in the secondary use of health data?

- What types of negative consequences come to your mind?

  For whom or what are these negative consequences relevant?

e.g. for healthcare providers, health services research (e.g. if data is not made available), policy on secondary use, etc.

- Based on the possible consequences, how sever do you consider these risks to be? / What relevance do these risks have when it comes to realising secondary use projects?

Refers to: willingness to share data, approval of reuse request, data sharing

- How do you assess the significance/relevance of these risks compared to the faclitating interests you mentioned earlier?

Part 3: Risk mitigation strategies/risk management

1. How do you assess the legal conditions for protecting the interests of service providers and of research with health data?

e.g. in the context of MII, but also other initiatives (e.g. NUM), secondary use of DRG routine data, of statutory quality assurance

2. Which statutes or subsidiary legislations/strategies already exist that can help minimising potential risks but at the same time promote the secondary use of health data?
   - How do you assess the effectiveness of these strategies?
   - From your perspective, are there any particularly effective strategies among them?
3. From your perspective, what additional strategies are needed in future to protect the interests of healthcare providers?
   - Against the background of the legal and technical developments, which strategies may need to be developed and implemented in future?
   - Are you aware of any good-practice strategies, possibly from abroad?
   - Potential strategy: Should potential interests/risks be explicitly taken into account in decision-making processes on secondary use requests in UACs?

Note: So far there are no specific review criteria included in use and access policies; only recommendations (e.g. scientific quality & objectives, expected added value, originality)

Closing questions

1. Is there anything we haven`t addressed that is of importance for you? Did we miss anything?

2. Do you think of any other persons with expertise in the topic that we should additionally invite?

End

- Thank you for your participation in this interview.
- Explain: Next steps in the project & further data processing
- Ask: Permission to approach for further questions, additions, etc.

Supplement 5 – Interview guide (German Original)

Studie: **Provider Interests in the Secondary Use of Health Data**
Interviewleitfaden – Stakeholder Interviews

Einleitung

Vorstellung der Interviewer*innen & Projekt (s. Hintergrundinformationen)

Fragen?

Einverständniserklärung prüfen – über **Aufnahme** informieren

Teil 1: Einstieg – Vorstellung und Eröffnungsfrage

2. Könnten Sie uns bitte die Schwerpunkte Ihrer Arbeit schildern und inwiefern die Sekundärnutzung von Gesundheitsdaten darin eine Rolle spielt?
    - Welche Erfahrungen haben Sie vor diesem Hintergrund mit der Sekundärnutzung von Gesundheitsdaten gemacht?
3. Zunächst ganz allgemein: Welche Interessen von Leistungserbringern werden aus Ihrer Perspektive durch die Sekundärnutzung von Gesundheitsdaten berührt?
    - Was könnten Interessen sein, die sich förderlich/hemmend auf eine solche Sekundärnutzung auswirken?

Teil 2: Risiken, ihre Merkmale, Folgen & Faktoren der Sekundärforschung

5. Wie schätzen Sie das ein: kann die Sekundärnutzung von Gesundheitsdaten mit Risiken für Leistungserbringer verbunden sein?
    - Können Sie Ihre Einschätzung bitte näher erläutern? / Wie kommen Sie zu der Einschätzung, dass die Sekundärnutzung (keine/geringe/hohe) Risiken birgt?

6. Was sind das aus Ihrer Sicht konkret für Risiken, die mit der Sekundärnutzung von Gesundheitsdaten für Leistungserbringer verbunden ist?
    - An welchen Punkten der Sekundärnutzung entstehen diese Risiken entstehen und wie?

    > Gemeint ist: Datenanfrage/-prüfung, Datenzugriff, -verarbeitung/-analyse, Publikation

    - Inwieweit unterscheiden sich solche Risiken je nach Art der involvierten Leistungserbringer (z.B. stationär vs. ambulant)?

7. Inwieweit haben die Art und der Kontext von Sekundärnutzungsprojekten/Forschungsfragen Einfluss auf die von Ihnen genannten Risiken?

    > ggf. Hinweise geben: Auf Basis vorheriger Diskussionen in Forschergruppe/Literaturrecherche mögliche Risiken:
    > **Art der Daten** und des Datenzugriffs (z.B. anonymisiert/pseudonymisiert; aggregiert vs. Einzeldaten)
    > **Forschungsziel** (z.B. risikobehaftet, einrichtungsintern ähnliche Vorhaben geplant)
    > **Person/Partei**, die die Daten anfordert (rechtlicher Status [z.B. Forschungseinrichtung/Privatunternehmen gewinnorientiert], Reputation, Herkunft, weitere beteiligte Parteien)

    - Können Sie [genannte Merkmale] näher erläutern? Welche Faktoren konkret machen das Risiko solcher Projekte/Forschungsfragen aus?

8. Welche negativen Folgen könnten resultieren, wenn solche Risiken in der Sekundärnutzung von Gesundheitsdaten nicht hinreichend adressiert werden?
   - Welche Arten von negativen Folgen kommen Ihnen in den Sinn?

     Für wen oder was sind diese negativen Folgen relevant?

     bspw. für Leistungserbringer, Versorgungsforschung (u.a. wenn Daten nicht zur Verfügung gestellt werden), Politik zur Sekundärdatennutzung, etc.
   - Als wie gravierend schätzen Sie, ausgehend von den möglichen Folgen, diese Risiken ein? / Welche Relevanz haben diese Risiken, wenn es um die Verwirklichung von Sekundärnutzungsprojekten geht?

     Gemeint ist: Bereitschaft zum Datenteilen, Genehmigung, Datenbereitstellung
   - Wie beurteilen Sie die Bedeutung/Relevanz dieser Risiken gegenüber den von Ihnen zuvor erwähnten förderlichen Interessen?

Teil 3: Strategien zur Eindämmung/zum Management von Risiken

4. Wie beurteilen Sie die rechtlichen Rahmenbedingungen zum Schutz von Interessen der Leistungserbringer und der Forschung mit Sekundärdaten?

   z.B. im Kontext der MI-I, aber auch anderen Initiativen (z.B. NUM), Sekundärnutzung von DRG-Routinedaten, der gesetzlichen Qualitätssicherung
5. Welche (unter-)gesetzlichen Strategien bestehen bereits, die mögliche Risiken minimieren aber die Sekundärnutzung von Gesundheitsdaten gleichzeitig ermöglicht?
   - Wie beurteilen Sie die Wirksamkeit dieser Strategien?
   - Gibt es Ihrer Einschätzung nach besonders effektive Strategien darunter?
6. Welche zusätzlichen Strategien zum Schutz der Interessen von Leistungserbringern braucht es aus Ihrer Perspektive zukünftig zusätzlich?
   - Welche Strategien müssen vor dem Hintergrund der rechtlichen und technischen Entwicklungen zukünftig ggf. erst entwickelt und implementiert werden?
   - Sind Ihnen good-practice Strategien, ggf. auch aus dem Ausland bekannt?
   - Mögliche Strategie: Sollten mögliche Interessen/Risiken explizit bei der Entscheidung über Sekundärnutzungsanträge in UACs berücksichtigt werden?

     Hinweis: bislang gibt es in der Nutzungsordnung keine spezifischen Prüfkriterien; lediglich Empfehlungen (z.B. wissenschaftliche Qualität & Ziele, zu erwartender Mehrwert, Originalität)

Abschließende Fragen

3. Was haben wir bislang nicht angesprochen, das Sie aber für bedeutsam halten? Haben wir irgendetwas übersehen?

4. Fallen Ihnen andere Person mit einschlägigem Wissen zum Thema oder Interessengruppen ein, deren Perspektive wir zusätzlich aufnehmen sollten?

Abschluss

- Vielen Dank, dass Sie an diesem Interview teilgenommen haben!
- Erläutern: Wie geht es weiter im Projekt & mit den Daten
- Frage nach Erlaubnis Kontaktierung im Fall von Fragen, Ergänzungen etc.
