# Supplementary material for: Benefits and risks of health data reuse for healthcare providers: stakeholder perspectives from a qualitative interview study
Source: BMC Health Serv Res. 2025 Mar 18;25:402. doi: 10.1186/s12913-025-12500-7 (PMC11917074; doi:10.1186/s12913-025-12500-7)
Supplement: Supplementary file 6 — Supplementary Material 6: Template Postscript [file 12913_2025_12500_MOESM6_ESM.pdf]

BIH QUEST
Center for Responsible Research

Postscript (English Translation)

Interview-ID	
Date	XX-XX 2023
Time | Duration	START – END| XX minutes
Location	Online
Participating Interviewers	

**Interview situation**	
**Information about interviewee**	
**Atmosphere (of conversation)**<br>• Relationship between interviewer and interviewee<br>• Development over the course of the interview<br>• Interaction	•
**Conversation before recording**	•
**Peculiarities/disruptions**	
**Conversation after recording**	•
**Central themes/ statements**<br><br>**Preliminary ideas for understanding/interpretations**	**Perceived interests**
	**Perceived risks | characteristics**
	**Influence of research questions | characteristics of secondary use projects**
	**Potential consequences & severity of risks**
	**Risk mitigation strategies | risk management**
	**Further information**
**Notes for further work**	

Protocol: [Name & Date]
Additions: [Name & Date]

Postscript (German Original)

Interview-ID	
Datum	XX.XX. 2023
Uhrzeit | Dauer	Start – Ende | XX Minuten
Ort	Online
Teilnehmende Interviewer*innen	

**Interviewsituation**	
**Informationen zum/zur Interviewpartner*in**	
**(Gesprächs-)Atmosphäre**<br>• Beziehung zwischen Interviewer*in – Interviewpartner*in<br>• Entwicklung des Gesprächsverlaufs;<br>• Interaktion	-
**Gespräche vor Aufnahme**	
**Besonderheiten/Störungen**	
**Gespräche nach Aufnahme**	
**Zentrale Themen | Aussagen**<br><br>**Erste Ideen zum Verständnis | für die Interpretation**	**Wahrgenommene Interessen**
	**wahrgenommene Herausforderungen & Risiken | Charakteristika**
	**Einfluss von Merkmalen von Sekundärnutzungsprojekten | Forschungsfragen**<br><br>-
	**Mögliche Folgen & Schwere der Risiken**<br><br>-
	**Strategien Eindämmung | Management von Risiken**<br><br>-
	**Weitere Informationen**<br><br>-
**Für die Weiterarbeit berücksichtigen**	-

Protokoll: [Wer hat erstellt & Datum]
Ergänzung: [Wer hat erstellt & Datum]
