# Supplementary material for: Benefits and risks of health data reuse for healthcare providers: stakeholder perspectives from a qualitative interview study
Source: BMC Health Serv Res. 2025 Mar 18;25:402. doi: 10.1186/s12913-025-12500-7 (PMC11917074; doi:10.1186/s12913-025-12500-7)
Supplement: Supplementary file 7 — Supplementary Material 7: Final coding tree [file 12913_2025_12500_MOESM7_ESM.pdf]

BIH QUEST
Center for Responsible Research

Supplement 7 – Coding Tree (English translation)

Note:

- Main categories (bold): deductive definition
- Subcategories (several levels): inductive development

**Coding tree**
**Potentials of secondary use**
Scope of statements: specific healthcare providers
Improve (quality of) healthcare
Improve transparency of healthcare
Improve research
Closing knowledge gaps
**Perceived risks of secondary use & their consequences**
Causes of risks
Data sources: lack of validity & systematic bias
Methodological challenges in the secondary use
Methodological expertise
Analytical approaches
Questions legal certainty
Loss of control over shared data and reuse purposes
Improving transparency in healthcare
Risks
Biased representation/interpretation of healthcare
Reuse purposes: lacking welfare orientation
Liability risk
Disclosure of challenges & misconduct in healthcare
Consequences of risk
Consequences at the micro level
Competitive/economic & existential harm
Reputational damage

Consequences at the meso and macro level
Loss of confidence in research with secondary data
Loss of healthcare structure
Healthcare management against particular interests
(Political) miscovernance of healthcare based on secondary data
**Risk mitigation: Context factors & strategies**
Appropriate management of use and access
Assessment of status quo
Adequate
Need for improvement
Risk mitigating use and access policies
Systematic & transparent interest- and risk-benefit assessment
Decision-making bodies: involve relevant stakeholders & ensure transparency
Establish transparency about access to and use of secondary data
Composition of decision-making bodies
Anchoring of decision-making bodies
Decentralised
National
Improve validity in health data reuse
Assessment of status quo
Adequate
Need for improvement
Improve interoperability, findability linkage of data sources
Improve data sources & data quality
Ensure research quality
Improve context conditions for secondary use
Assessment of status quo
Adequate
Need for improvement
Develop goals and strategies for secondary use

Ensure legal certainty
Ensure data security
Promote trust and acceptance
Assessment of status quo
Adequate
Need for improvement
Among healthcare providers
In the population

Supplement 7 – Coding Tree (German Original)

Hinweise:

- Hauptkategorien (fett): deduktive Anwendung
- Thematische Subkategorien (mehrere Ebenen): induktive Entwicklung

**Codesystem**			
**Potenziale der SD-Nutzung**			
	Geltungsbereich der Aussagen: spezifische Leistungserbringer		
	Versorgung(-squalität) verbessern		
	Transparenz über Gesundheitsversorgung verbessern		
	Verbesserung Forschung		
	Erkenntnislücken schließen		
**wahrgenommene Risiken der SD-Nutzung & Folgen**			
	Ursachen für Risiken		
		Datenquellen: mangelnde Validität & systematische Verzerrungen	
		Methodische Herausforderungen in der SD-Nutzung	
			Methodenkompetenz
			analytische Verfahren
		Fragen Rechtssicherheit	
		Kontrollverlust Datennutzung & -weitergabe	
		Verbesserung Transparenz in der Versorgung	
	Risiken		
		Verzerrte Abbildung/Interpretation der Vers.-Realität	
		Nutzungszwecke: Mangelnde Gemeinwohlorientierung	
		Haftungsrisiko	
		Offenlegung von Herausforderungen Versorgung & Fehlverhalten	
	Risikofolgen		
		Folgen auf der Mikroebene	
			Wettbewerbliche/wirtschaftliche & existenzielle Schäden
			Reputationsschädigung

- Folgen auf der Meso- und Makroebene
  - Vertrauensverlust in die Forschung mit Sekundärdaten
  - Wegfall Versorgungsangebot
  - Versorgungssteuerung entgegen Partikularinteressen
  - (pol.) Fehlsteuerung der Versorgung auf Basis SD
- **Rahmenbedingungen & Strategien Risikomanagement SD-Nutzung**
  - angemessene Steuerung Zugang und Nutzung SD
    - Einschätzung Status quo
      - angemessen
      - verbesserungswürdig
    - Risikominimierende Zugangs- und Nutzungsregeln
    - Systematische & transparente Interessen-/Risiko-Nutzen-Abwägung
    - Entscheidungsgremien: Stakeholder einbeziehen & Transparenz herstellen
      - Transparenz über Zugang und Nutzung SD herstellen
      - Zusammensetzung von Entscheidungsgremien
      - Verankerung von Entscheidungsgremien
        - Dezentral
        - National
  - Validität in der SD-Nutzung verbessern
    - Einschätzung Status quo
      - angemessen
      - verbesserungswürdig
    - Interoperabilität, Auffindbarkeit & Linkage von Datenquellen verbessern
    - Datenbasis und -qualität verbessern
    - Forschungsqualität sicherstellen
  - Rahmenbedingungen der SD-Nutzung verbessern
    - Einschätzung Status quo
      - angemessen
      - verbesserungswürdig
    - Ziele und Strategien SD-Nutzung erarbeiten

	Rechtssicherheit gewährleisten
	Datensicherheit gewährleisten
	Akzeptanz und Vertrauen fördern
	Einschätzung Status quo
	angemessen
	verbesserungswürdig
	bei Leistungserbringern
	in der Bevölkerung
